# Supplementary material for: Physiological response of the cold-water coral Desmophyllum dianthus to thermal stress and ocean acidification
Source: PeerJ. 2016 Feb 2;4:e1606. doi: 10.7717/peerj.1606 (PMC4741066; doi:10.7717/peerj.1606)
Supplement: Table S1 — Temperature, salinity, pH, alkalinity and DIC (dissolved inorganic carbon) were measured. All other values (pCO2; Ωaragonite, aragonite saturation state; Ωcalcite, calcite saturation state) were calculated using CO2 calc (Robbins et al., 2010) from pH, alkalinity and DIC. [file peerj-04-1606-s002.docx]

**Supplementary Table 1**: Chemical and physical properties of seawater systems (mean  ±  1 SD) from October 2014 to July 2015. Temperature, salinity, pH, alkalinity and DIC (dissolved inorganic carbon) were measured. All other values (pCO_2_; Ω_aragonite_: aragonite saturation state; Ω_calcite_: calcite saturation state) were calculated using CO_2_calc ([Robbins et al., 2010](http://www.sciencedirect.com/science/article/pii/S0025326X12005619#b0395)) from pH, alkalinity and DIC.

| Target temperature and pCO_2_ | | 9 °C, 390 ppm | 9 °C, 750 ppm | 12 °C, 390 ppm | 12 °C, 750 ppm |
| --- | --- | --- | --- | --- | --- |
| Recorded values | Temperature (°C) | 9.12 ± 0.30 | 9.69 ± 0.34 | 12.33 ± 0.17 | 12.26 ± 0.19 |
|  | Salinity (psu) | 38.51 ± 0.33 | 38.16 ± 0.33 | 38.49 ± 0.43 | 38.24 ± 0.49 |
|  | pH | 7.93 ± 0.12 | 7.79 ± 0.16 | 7.89 ± 0.13 | 7.75 ± 0.15 |
|  | Alkalinity (mmol l^-1^) | 2.58 ± 0.06 | 2.56 ± 0.04 | 2.47 ± 0.07 | 2.48 ± 0.09 |
|  | DIC (μmol kg^−1^) | 2423.29 ± 75.82 | 2448.44 ± 74.42 | 2305.20 ± 91.99 | 2376.80 ± 89.66 |
|  | pCO_2_ | 612.49 ± 202.15 | 879.38 ± 360.87 | 651.38 ± 216.78 | 947.16 ± 336.72 |
|  | Ω_calcite_ | 2.98 ± 0.67 | 2.33 ± 0.80 | 2.95 ± 0.72 | 2.24 ± 0.76 |
|  | Ω_aragonite_ | 1.90 ± 0.43 | 1.49 ± 0.51 | 1.89 ± 0.46 | 1.44 ± 0.49 |
